# Musical, language, and reading abilities in early Portuguese readers

**DOI:** 10.3389/fpsyg.2013.00288

**Published:** 2013-06-18

**Authors:** Jennifer Zuk, Paulo E. Andrade, Olga V. C. A. Andrade, Martin Gardiner, Nadine Gaab

**Affiliations:** ^1^Laboratories of Cognitive Neuroscience, Developmental Medicine Center, Boston Children's HospitalBoston, MA, USA; ^2^Department of Pedagogical Studies, School of Elementary and Secondary Education, Colégio CriativoMarília, São Paulo, Brazil; ^3^Center for the Study of Human Development, Brown UniversityProvidence, RI, USA; ^4^Harvard Medical SchoolBoston, MA, USA; ^5^Harvard Graduate School of EducationCambridge, MA, USA

**Keywords:** Music, Language, Reading in Children

## Abstract

Early language and reading abilities have been shown to correlate with a variety of musical skills and elements of music perception in children. It has also been shown that reading impaired children can show difficulties with music perception. However, it is still unclear to what extent different aspects of music perception are associated with language and reading abilities. Here we investigated the relationship between cognitive-linguistic abilities and a music discrimination task that preserves an ecologically valid musical experience. 43 Portuguese-speaking students from an elementary school in Brazil participated in this study. Children completed a comprehensive cognitive-linguistic battery of assessments. The music task was presented live in the music classroom, and children were asked to code sequences of four sounds on the guitar. Results show a strong relationship between performance on the music task and a number of linguistic variables. A principle component analysis of the cognitive-linguistic battery revealed that the strongest component (Prin1) accounted for 33% of the variance and Prin1 was significantly related to the music task. Highest loadings on Prin1 were found for reading measures such as Reading Speed and Reading Accuracy. Interestingly, 22 children recorded responses for more than four sounds within a trial on the music task, which was classified as Superfluous Responses (SR). SR was negatively correlated with a variety of linguistic variables and showed a negative correlation with Prin1. When analyzing children with and without SR separately, only children with SR showed a significant correlation between Prin1 and the music task. Our results provide implications for the use of an ecologically valid music-based screening tool for the early identification of reading disabilities in a classroom setting.

## Introduction

Processing both music and language require similar perceptual and cognitive processes within and outside of the auditory domain including the processing of pitch and rhythm, rapid auditory processing, selective attention, or working memory. Musicianship has been shown to improve or correlate positively with language and literacy skills in numerous areas such as phonological awareness (Lamb and Gregory, 1993; Anvari et al., 2002; Burnham and Brooker, 2002; Montague, 2002; Peynircioglu et al., 2002; Overy, 2003; Norton et al., 2005; David et al., 2007; Foregeard et al., 2008; Holliman et al., 2010; Dege and Schwarzer, 2011; Moritz et al., 2012), second-language phonological ability (Slevc and Miyake, 2006), and reading ability (Hurwitz et al., 1975; Barwick et al., 1989; Lamb and Gregory, 1993; Douglas and Willatts, 1994; Gardiner et al., 1996; Standley and Hughes, 1997; Anvari et al., 2002; Register et al., 2007). Most of these studies have been conducted with school-age or adult subjects but positive relationships between musical ability/activity and underlying component skills of reading have also been reported before reading instruction starts (e.g., Fisher and McDonald, 2001; Moritz et al., 2012). Several studies report a strong relationship between musical training and language processing in children and adults using brain imaging techniques (e.g., Jentschke et al., 2005; Moreno and Besson, 2006; Wong et al., 2007). For example, individuals with extensive musical training demonstrate enhanced speech perception abilities, specifically in pitch processing of speech sounds (Schon et al., 2004) and the neural coding of speech (e.g., Musacchia et al., 2007, 2008; Kraus et al., 2009; Parbery-Clark et al., 2012). Improved speech abilities have been demonstrated in children following 6 months of musical training (Moreno et al., 2009) and longitudinal improvements in speech segmentation and pre-attentive processing of syllabic duration have been shown following 2 years of musical training compared to a control painting program (Chobert et al., 2012; Francois et al., 2012). Furthermore, musicians have demonstrated heightened ability over non-musicians in cognitive processes such as tonal and verbal working memory (Chan et al., 1998; Kilgour et al., 2000; Ho et al., 2003; Franklin et al., 2008; Schulze et al., 2011) and subcortical processing of speech has also been found to align with reading and music aptitude in children (Strait et al., 2011).

Most children learn to read effortlessly within the first 2 years of reading instruction, but about 5–17% of children struggle despite normal hearing and vision and adequate instruction (Snowling et al., 2003). To date, children with a reading disability (RD) cannot be reliably diagnosed until second or third grade following demonstrated failure with literacy acquisition, which can have severe social and psychological consequences. Identification of children at risk earlier on offers the chance for individualized attention, implementation of interventions shown to be helpful, and amelioration of later struggles. Although behavioral risk factors for a RD can be identified in preschool (Scarborough, 1998), reliance on behavioral assessments alone can produce a relatively high rate of false-positives (Badian, 1994; Frith, 1999) as well as some false-negatives (Coleman and Dover, 1993; O'Connor and Jenkins, 1999; Compton et al., 2006), which can lead to poor allocation of limited funds for interventions (Compton et al., 2010; McNamara et al., 2011). Additionally, these assessments are not available for all cultures and languages, and are not necessarily effective for second-language learners. A music-based screening tool offers the potential for a convenient, engaging screening measure that can be implemented in school settings lacking other resources, to identify children at risk for language difficulties early on.

Several screening batteries for the early identification of children at risk in preschool or kindergarten have been examined and findings suggest a range of cognitive, linguistic, and basic auditory processing impairments in preschoolers who later exhibit weak reading scores. These include phonological processing (e.g., Nation and Hulme, 1997; Pennington and Lefly, 2001; Snowling et al., 2003; Flax et al., 2009), and speech perception, production, and comprehension (Pennington and Lefly, 2001; Benasich and Tallal, 2002; Flax et al., 2009). Phonological processing weaknesses in struggling readers typically present challenges with rhyming, alliteration, or the identification of subtle similarities and changes between words presented aurally (Bradley and Bryant, 1978). Additionally, individuals with a RD often present temporal auditory discrimination deficits in the linguistic and non-linguistic domain (Tallal and Piercy, 1973; Temple et al., 2000; Tallal, 2004). Extensive auditory training in the temporal domain has demonstrated improved language and reading abilities in children and adults with a RD (Temple et al., 2003; Gaab et al., 2007). Some evidence also points to a rhythmic perception deficit in individuals with RD (Muneaux et al., 2004; Thomson and Goswami, 2008). Rhythm production has been shown to be weaker in children and young adults with RD compared to typical readers (Wolf et al., 2002; Thomson et al., 2006). Similarly, metrical perception (discrimination of metrical structure, i.e., beat frequency and musical accent within two rhythmic phrases) relates significantly to development in typical children and those with RD (Huss et al., 2011). Furthermore, music-based intervention in struggling readers suggests that music can be used as a remediation tool for improving spelling and reading (Atterbury, 1985; Farmer et al., 1995; Overy, 2003; Santos et al., 2007). This combined evidence posits that specific musical training may be an effective outlet for reading remediation and/or the identification of children with RD (Tallal and Gaab, 2006).

A putative relationship between musical perception and reading ability has been established, but it is still unclear what types of musical stimuli (e.g., tonal, rhythmic) specifically relate to literacy skills, and whether a music-based assessment could be used as a diagnostic tool for the early identification of children with language and/or reading difficulties. Music perception has been previously quantified through a variety of dependent variables including pitch (Jusczyk and Krumhansl, 1993; Moreno et al., 2009), rhythm and meter (Palmer and Krumhansl, 1987; Douglas and Willatts, 1994; Anvari et al., 2002; Montague, 2002; Holliman et al., 2010; Huss et al., 2011; Moritz et al., 2012), melody discrimination (Overy, 2003), and musical expectancy (Bharucha and Stoekig, 1986). Perceptual tasks have also been designed specifically for non-musical preliterate children and early readers, such as perception of musical meter (Goswami et al., 2013), implicit chord processing (Tillman et al., 2000), pitch processing (Moreno et al., 2009), and tonality sensitivity (Gerry et al., 2012). Musical tasks in most previous research studies have utilized musical stimuli through keyboard recordings (Overy, 2003), vocal recordings (Besson et al., 2007), or computer-generated sounds (Overy, 2003; Gaab et al., 2005), presumably administered in a laboratory setting. The present study seeks to investigate whether the relationship between musical perception and reading ability holds for a music discrimination task comprised of isochronous sequencing and discrimination of chords that additionally preserves the ecological validity of the musical experience. Music requires concurrent spatial and temporal processing, encompassing auditory sequencing demands similar to those necessary in language: processing and integration of timing, order, relations, and components of phrases or sentences (Janata and Grafton, 2003; Zatorre et al., 2007; Tillman, 2012). Our music task has been specifically developed to capture this ability in beginning readers. It was designed to preferentially engage perceptual and cognitive mechanisms dedicated to “auditory pattern sequencing,” including auditory working memory but also other operations such as the mapping of perceived sounds onto written symbols as well as a decision-making component followed by a subsequent motor response. Additionally, our task maintains an ecologically valid musical experience and is a convenient tool that can be easily administered by an educator in a classroom setting, especially in educational settings lacking resources and expertise for the early identification of children at risk for language and/or reading disabilities. Therefore, in the current study we aim to evaluate the relationship between performance on our music task and the cognitive-linguistic capacities required for reading as well as reading skills *per se*, to further elucidate the specificity and sensitivity of music perception tasks for language and reading ability.

## Materials and methods

### Participants

45 students (29 male; 14 female) were selected to participate in this study from *“Colégio Criativo,” Marília*, an elementary school in São Paulo, Brazil. Consent from parents was obtained before participation. All testing occurred during class time with permission from the school administration, principal, and teachers. Students recruited for the study were in the second grade of primary school, as per the grade distinctions in the Brazilian education system. Age was calculated at the onset of test administration, and students ranged in age from 6 to 8 years (average: 7 years and 2 months, SD: 4 months). 41 students out of 45 were right-handed. Two students were excluded due to deficits in recalling all letters of the alphabet, resulting in a disadvantage for our reading and writing tasks. All participants had normal hearing, no speech deficits (e.g., stuttering), and no musical training. All the participants were native speakers of Brazilian Portuguese, the language required of all test administration and tasks in the study. All students came from upper-middle class families and most had at least one parent who was a working professional.

### Behavioral measures

#### Psychometric measures

Linguistic and cognitive abilities of all participants were evaluated by administering tasks from the Cognitive-Linguistic Protocol by Capellini and Smythe (2008). Developed in Brazil, the purpose of this protocol is to identify the cognitive-linguistic profile in the first stages of reading acquisition and assess the cognitive-linguistic abilities of Brazilian children. This protocol was the best measure of these abilities available to test administrators in Brazil and designed in the native language, Portuguese. Specific measures used and scoring criteria are outlined below:

#### Alphabet task

Students were asked to recall all the letters of the alphabet in written form. There was no prompting or assistance. Scores were out of 26: the number of correct letters the child wrote down. The task was untimed.

#### Reading tasks

- Reading speed: number of words read aloud by the student in 1 min from a list of 70 words. Students are instructed to read each word audibly, start with the first line and then continue directly to the next. The maximum possible score was 70.
- Reading accuracy: number of words read aloud correctly from a list of 70 words. This task was scored within the same trial as reading speed, with a maximum score of 70.
- Reading completion: time required for student to read the entire list of 70 words within the same trial as reading speed and reading accuracy. Once the child passed 1 min reading aloud (for reading speed), timing continued until they complete the list of 70 items. Time was recorded in seconds.
- Reading pseudowords: number of pseudo words read aloud correctly from a list of 10. Maximum score was 10. Task was untimed.

#### Writing tasks

- Writing words: number of words written with correct spelling from a list of 30 presented aurally. Maximum score of 30. Task was untimed.
- Writing pseudowords: number of pseudowords written with correct spelling from a list of 10 presented aurally. Maximum score of 10. Task was untimed.

#### Phonological tasks

- Alliteration: number of correctly identified two words with the same initial sound out of a total list of three words spoken by an examiner. There were 10 trials, leading to a maximum score of 10.
- Rhyme detection: number of correctly identified two words with the same ending out of a total list of three words spoken by examiner. There were 20 trials, leading to a maximum score of 20.
- Syllable segmentation: students were asked to repeat the word spoken by the examiner while tapping to each syllable. Number of correct syllable breakdowns leading to a maximum possible score of 12.
- Auditory word discrimination: number of correct identifications of whether two heard words, spoken by the examiner, are the same or different. Words can differ by only one phoneme. Maximum possible score of 19 for 19 trials.

#### Rhythm production task

Number of correctly reproduced rhythmic items following demonstrations by the experimenter (with rhythms tapped on the desk). Rhythm items increase in length throughout the task, extending from 2 up to 10 taps with the pencil. Maximum possible score of 12 (one point for each trial) for correct rhythm production on each trial.

#### Verbal working memory

- Word sequence: number of correctly repeated two to five-word sequences (two unique trials for each sequence with the exception of the first example) spoken by the examiner in an interstimulus interval of 1 s. Familiar two or three-syllable words were presented. Score was given by the number of sequences correctly accomplished, with a maximum score of 7.
- Pseudoword repetition: number of correctly repeated pseudo words from a list of 23 presented aurally by the examiner. Maximum score of 23 for pseudowords spoken correctly.
- Verbal number sequence backwards: number of correctly repeated two to five-number sequences backwards (two trials for each sequence) that were spoken by the examiner. Maximum score of 8.

#### Shapes copying

Correctly copied archetypal forms. Task included four shapes: a circle, a square, a diamond, and a complex abstract figure. Students were able to see shapes while copying was performed. Students were only allowed to erase work on the last shape. Figures were scored through comparison with a standardized table of different visual representations of the shapes and measured with a diagramed seven-point scale. Maximum score for the task was 7.

#### Visual short term memory

- Figure order: ordering of solid abstract figures, i.e., after seeing two to five-figure sequences over a 10 s period, student was asked to reassemble the ordered pattern of figures in the same order and rotation. Score is given by the number of sequences in which the figures were correctly ordered, with a maximum score of 8.
- Figure rotation error: number of errors made in rotation of the shapes in figure order task. 28 figures were presented throughout the sequences, leading to a maximum of 28 errors.

#### Rapid identification

- Rapid figure identification: student was asked to rapidly name a list of four objects (house, ball, elephant, clock) displayed pictorially in a particular order. A different ordering was presented in each trial, with 10 total trials. Total time was recorded in seconds.
- Rapid number identification: student was asked to rapidly name numbers one through nine as listed visually in a random order. Screening was performed before task to confirm that the child knew numbers one through nine. Time was recorded in seconds.

#### Musical sequence transcription task

**Figure 1:**
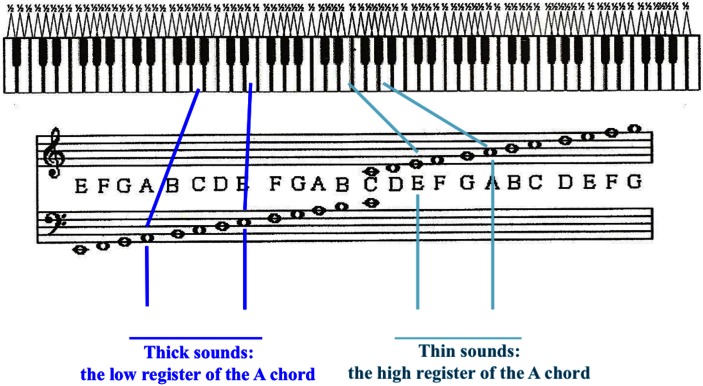
**Musical notation and octaves on piano of notes played on the guitar in MSTT**. Blue lines indicate notes in “thick sound;” teal lines indicate “thin sound.”

***Design of task.*** A sequential and isochronous music measure was used, developed by Paulo Estêvão Andrade specifically for this study. Musical Sequence Transcription Task (MSTT) was designed to preferentially engage perceptual and cognitive mechanisms dedicated to “auditory pattern sequencing” including auditory working memory but also involves other operations, such as the mapping of perceived sounds onto written symbols as well as a decision-making component followed by a subsequent motor response. The music task involved a sequence of four two-note chords played in rhythmic sequence on the guitar in predetermined arrangements. Two chords were presented: one in the low register and the other in the high register of the instrument. Translated from Portuguese, the students were taught to code these chords as the “thick sound” for the low, and “thin sound” for the higher. The chords also differed in the musical intervals that they displayed. The lower register chord used an interval of a perfect fifth, with fundamental frequencies 110 (A) and 165 Hz (E). The higher register chord used an interval composed of a perfect fourth, with 330 (E) and 440 Hz (A). Thus both intervals included the same pitches, A and E, but span two octaves between the low A of the “thick sound” and the high A of the “thin sound.” A difference in interval quality (perfect fourth and perfect fifth) between the two stimuli was necessary to avoid detection of overtones in the “thick sound,” leading to a perceived higher, “thin sound.” Visualization of pitches in the stimuli on the piano and musical staff is shown in Figure 1. MSTT was designed in such a way that it avoids fine perception of pitch variations. We wanted to ensure that a low performance on MSTT could not be explained by a possible low level deficit in the fine-grained pitch analysis (less than two semitones) that characterizes a subject with congenital amusia (e.g., Loui et al., 2011).

***Administration of the musical sequence transcription task.*** Students were first introduced to the two musical sounds and trained and guided in distinguishing between the two. Participants were provided repeated exposure to both of the sounds used in the sequences and encouraged to verbalize and discuss the differences between them. As part of this process, each sound was introduced with the specific descriptive names “thick” and “thin.” The names were chosen based on the particular attributes that students had attributed to the sounds in discussion. These monikers help prevent mistakes based purely on a student's confusion with naming a sound as opposed to confusion related to distinguishing the sound. All students were given the same amount of discussion time, trials, and exposure to the two sounds. Sounds in MSTT were coded into symbols that students were instructed to record throughout the task. The thin sound (the higher pitched fourth) was marked with a “|” and the thick sound (the lower pitched fifth) was marked by an **“O.”**

Sequences of the MSTT were presented to participants in a slow, isochronous manner, consistent in tempo throughout the entire task (approximately 88 beats per min). The administrator played all four-sound sequences with the frets of the guitar facing away from the students, to prevent participants from using visual cues to guess the sounds being played. After a short pause equal to the length of the sequence, students were asked to record the sounds in the order presented using the symbol system for the “thin” and “thick” sounds. Children were never explicitly informed that there would be four sounds in each sequence. Students were not permitted to write anything prior to a signal from the administrator. The entire task comprised of 20 sequences, each sequence being a four-sound predetermined arrangement of the two possible sounds (thick or thin). MSTT comprised of nine unique sequences, presented and then repeated in the same order with an additional two repetitions of the first sequence presented halfway through each repetition of patterns, comprising 20 trials total.

***Scoring criteria***. Each sequence was scored individually, leading to a maximum score of 20 for the task. A correct response for each sequence was achieved by accurate handwritten recording of the four sounds of the sequence in the same order as presented using the symbol system for the “thin” and “thick” sounds described. When less than four sounds were marked for a particular trial, even if partially correct, the trial was incomplete, was not given credit, and thereby scored as zero. This was considered as an incidence of Insufficient Responses (IR). If more than four sounds were recorded for a trial, this was also considered incorrect and scored as zero for the trial. This reporting of more than four sounds was labeled as an incidence of Superfluous Responses (SR). The SR score was also determined as the total number of trials on which SR had appeared, leading to a maximum score of 20 if the participant had produced SR on every trial.

### Procedure

All participants were tested on MSTT and all tasks from The Cognitive-Linguistic Protocol by Capellini and Smythe (C&S). MSTT was administered to all participants concurrently in the music classroom, followed by individual and group administration of the linguistic and cognitive tests over the course of 6 weeks. The following assessments were administered individually: reading speed, reading accuracy, reading completion, reading pseudo-words, alliteration, rhyme, syllable segmentation, auditory word discrimination, rhythm production, word sequence, pseudoword repetition, verbal number sequence backwards, rapid figure identification, rapid number identification, figure order, and figure rotation error. By contrast, the following subtests were administered in the classroom: the Alphabet Task, Writing Words, and Writing Pseudowords. All study participation took place during school hours, so classroom administration was implemented for time efficiency on tests that did not require one-on-one monitoring. Testing began at the beginning of the academic calendar year.

### Statistical analysis

Mean scores were calculated for the entire group on each assessment across all participants. To investigate the extent and nature of relationships between MSTT and cognitive and linguistic assessments, Correlation Analysis (CA) and Principle Component Analysis (PCA) were implemented through SAS (SAS Institute, Inc., Cary, NC, USA). The analysis was designed to investigate both the individual and joint significant relationships between the musical and other variables. *t*-Tests were employed in order to examine differences between children with and without SR. Furthermore, we used partial Spearman CA to explore two hypotheses concerning contributions to the correlations of MSTT performance with scores on Prin1, and outcomes for subtests related to reading.

## Results

Mean scores and standard deviations were calculated for each measure of C&S cognitive and linguistic measures and MSTT performance, as shown in Table 1. Scoring of performance on MSTT revealed a large number of cases of what we term SR, i.e., more than four responses recorded on a single trial of MSTT, with 22 of the 43 participants displaying at least one occurrence of SR. No significant difference in age was found for children with versus without SR. As is outlined in Table 1, children with no occurrences of SR demonstrated significantly better performance than children with SR on a number of C&S measures, including Reading Speed (*t* = 2.872, *p* < 0.01), Writing Words (*t* = 2.221, *p* < 0.05), Auditory Word Discrimination (*t* = 2.167, *p* < 0.05), Word Sequence (*t* = 2.786, *p* < 0.01), and Shapes Copying (*t* = 2.398, *p* < 0.05). Children with no SR compared to those with additionally showed higher performance on Rhythm Production (*t* = 3.417, *p* < 0.005) and MSTT (*t* = 4.772, *p* < 0.0001). Additionally, 12 of the subjects showed performance patterns of what we term IR, i.e., less than four responses sounds recorded on a single trial. Eight of the IR subjects showed at least one case of SR as well.

**Table 1:** **Descriptive characteristics (mean ± standard deviation) of performance on cognitive-linguistic measures and MSTT in children who exhibited at least one occurrence of SR are compared to children with no SR**.

**Task**	**Children with no SR (*n* = **21**)**	**Children with SR (*n* = **22**)**	**p-Value**
	**Mean ± SD**	**Mean ± SD**	
Reading speed	39.52 ± 14.774	27.91 ± 11.625	0.006^**^
Reading accuracy	65.62 ± 11.478	62.41 ± 8.776	0.308
Reading completion	140.19 ± 115.348	180.73 ± 84.759	0.195
Reading pseudowords	9.57 ± 1.326	9.18 ± 1.259	0.329
Writing words	25.52 ± 4.622	22.32 ± 4.834	0.032^*^
Writing pseudowords	7.95 ± 2.179	6.68 ± 2.009	0.053
Alliteration	8.62 ± 1.687	7.86 ± 1.859	0.171
Rhyme detection	17.38 ± 3.008	15.77 ± 2.724	0.073
Syllable segmentation	11.76 ± 0.539	11.32 ± 1.041	0.089
Auditory word discrimination	18.95 ± 0.218	18.32 ± 1.323	0.036^*^
Rhythm production	6.1 ± 1.670	4.23 ± 1.901	0.001^**^
Word sequence	4.29 ± 1.007	3.41 ± 1.054	0.008^**^
Pseudoword repetition	20.9 ± 2.448	20.77 ± 1.798	0.841
Verbal number sequence backward	4.67 ± 1.560	4.32 ± 1.524	0.463
Shapes copying	5.62 ± 1.564	4.18 ± 2.281	0.021^*^
Figure order	5.76 ± 1.221	5.55 ± 1.101	0.545
Figure rotation error	2.38 ± 3.457	2.23 ± 2.844	0.874
Rapid figure identification	37.52 ± 8.875	38.55 ± 7.385	0.683
Rapid number identification	44.57 ± 10.708	43.14 ± 6.628	0.598
MSTT	15.29 ± 3.964	9.45 ± 4.044	0.001^**^

Asterisks indicate significance (

* p < 0.05,

** p < 0.01,

^***^p < 0.001).

Inspection of the data showed a few significant outliers to have excessive leverage distorting the further analysis. Rather than removing data from these subjects from the analysis, ranking transformations were employed to prevent a distorted influence on the findings. We also observed that in children without SR, some of the variables show ceiling effects. The further analyses described below should not be affected by this. Ceiling effects were observed in the following variables: reading accuracy, reading pseudowords, writing pseudowords, syllable segmentation, and auditory word discrimination.

### MSTT in relation to C&S linguistic and cognitive measures

After performing ranking transformation, Spearman CA was first used to identify which if any C&S measures showed a statistically significant relationship to MSTT. Thirteen cognitive and linguistic measures significantly related to MSTT: reading speed (*r* = 0.4411, *p* < 0.005), reading accuracy (*r* = 0.6026, *p* < 0.0001), reading completion (*r* = −0.3821, *p* < 0.05), reading pseudowords (*r* = 0.3065, *p* < 0.05), writing words (*r* = 0.4832, *p* < 0.005), writing pseudowords (*r* = 0.3515, *p* < 0.05), rhyme detection (*r* = 0.3372, *p* < 0.05), auditory word discrimination (*r* = 0.3826, *p* < 0.05), alliteration (*r* = 0.4075, *p* < 0.01), rhythm production (*r* = 0.4229, *p* < 0.005), word sequence (*r* = 0.4675, *p* < 0.005), shapes copying (*r* = 0.3276, *p* < 0.05), and figure order (*r* = 0.3728, *p* < 0.02).

In this paper we will focus our attention on an analysis, PCA, which takes account of the intercorrelations between performances on the subtests of the C&S measurement battery. This analysis first calculates a linear variable, called the first Principle Component, which weighs and linearly combines the C&S variables taking account of their intercorrelations so as to most efficiently characterize the variance of performance within all the variables of the C&S battery as a group. The weighting of individual variables reflects their relative contribution to Prin1. A second linear Principle Component variable then takes account for as much of the remaining variance within the C&S battery not yet accounted for by Prin1, a third takes account of as much variance as possible not accounted for by Prin1 and Prin2 and this continues until all the performance variability of the C&S battery is accounted for. No rotation was used to change projection of these computed variables onto the data set. We decided in advance to compute only as many Principle Component variables as needed to account for at least 70% of the C&S performance variance. We expected that only a small number of Principle Component variables would be needed to characterize the variability within the C&S. Thus if any of the Principle Component variables were found to show a relationship to music variables, the multiple-variables problem (i.e., the chance that this relationship was due to the number of variables whose relationship to the music variables were being measured) will be minimized.

We observed that the first variable computed by our PCA accounted for 33% of the C&S performance variation and that only five further variables were needed to account for more than 70% of the C&S variance. We then examined through CA the relationship of the score on each of these Principle component variables to the score on MSTT. Prin1 was significantly related to the score on MSTT (*r* = 0.5228, *p* < 0.0004). None of the other Principle Component variables included showed a significant relationship to MSTT. The relationship between Prin1 and MSTT remains highly significant (*p* < 0.001) after Bonferroni correction to account for multiple comparisons.

To begin to interpret Prin1, we look at its relationship to (i.e., loadings on) each of the C&S measurements. Highest Loadings are found for Reading Speed (0.3412), Reading Accuracy (0.3037), Reading Completion (0.3348), and Writing Words (0.332). Somewhat lower loadings are present for measures of performance on skills involving or contributing to but not fully measuring specifically full linguistic performance including reading pseudowords (0.2186), writing pseudowords (0.2070), alliteration (0.2692), rhyme detection (0.2492), and word sequence from our verbal working memory test (0.2341) and rapid figure naming (0.2657). Loading on other contributors to linguistic performance are still weaker, including performance on syllable segmentation (0.1928), word discrimination (0.1980), rhythmic production (0.1930), pseudoword repetition (0.1432), verbal number sequence backwards (0.1517), and rapid number identification (0.1689). The weakest loadings are on cognitive performances on skills least directly related to or least involving language. These included shapes copying (0.0918), figure order (0.101), and figure rotation error (0.1131). Thus, Prin1 seems to reflect a capability that is related to highest levels of linguistic performance but could also be explained by general auditory processing skills. It related most weakly to cognitive skills not specifically related to linguistic ability and lower cognitive processes that are not necessarily required during linguistic processing. Spearman Partial CA shows that after correcting for Prin1, the relationship of every individual C&S variable to MSTT is no longer significant after Bonferroni correction.

### Effect of superfluous responses on relationship of MSTT to PRIN1

**Figure 2:**
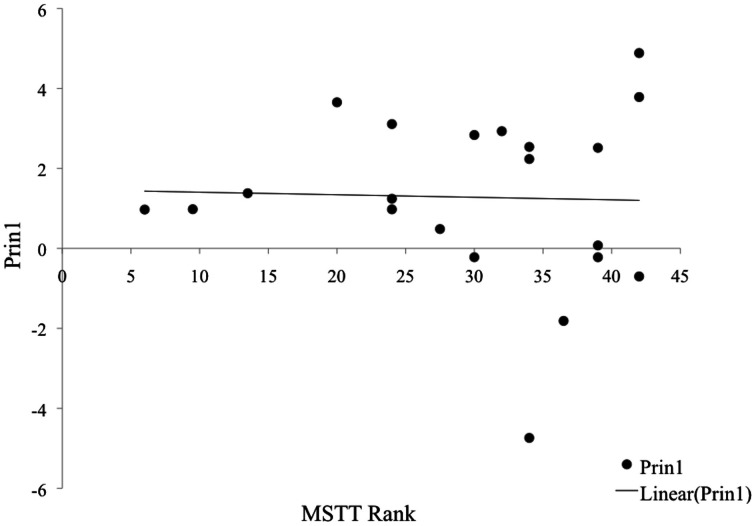
**Relationship of Prin 1 to MSTT in children with no SR or IR**.

**Figure 3:**
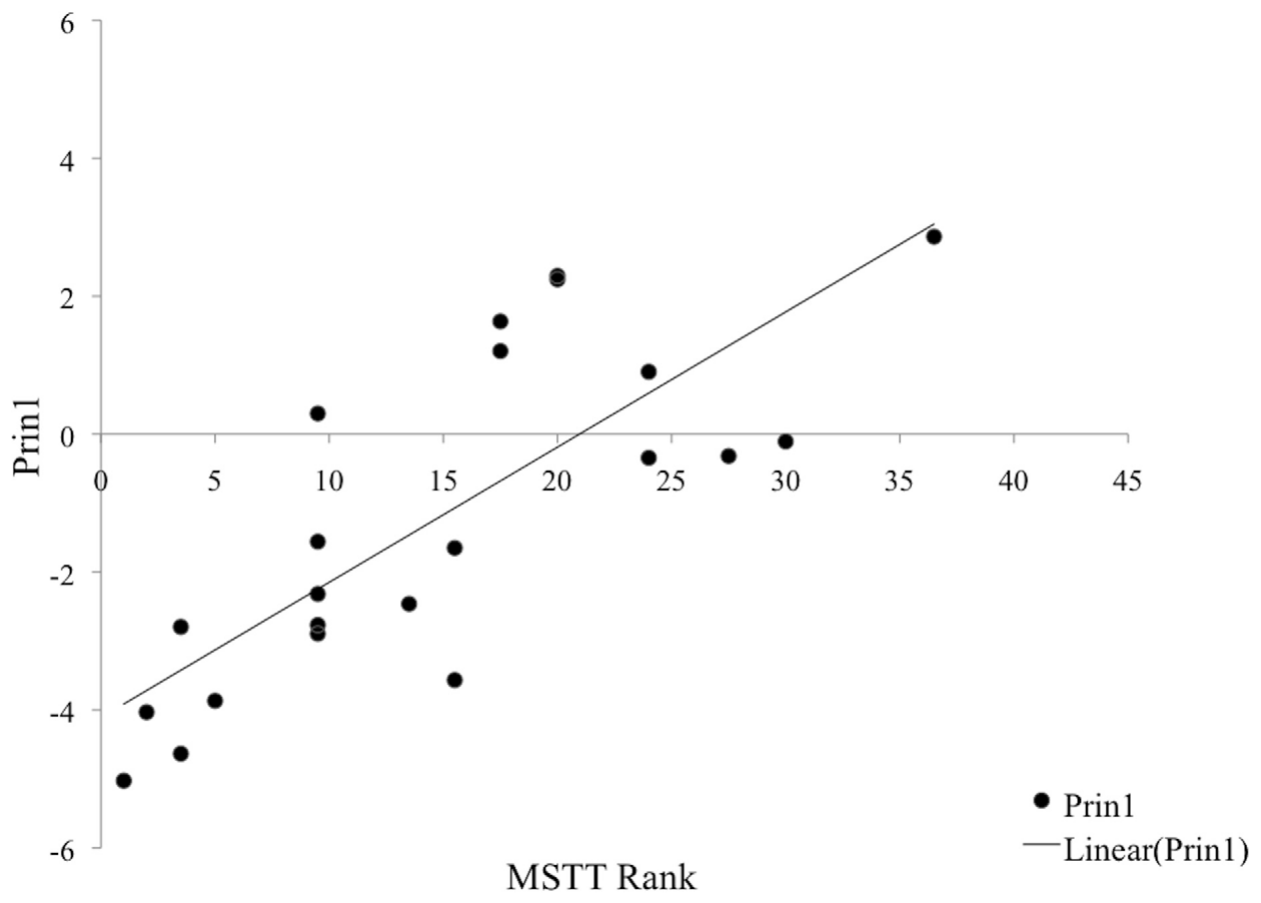
**Relationship of Prin1 to MSTT in children with SR**.

With such a significant occurrence of SR within MSTT performance, we examined how MSTT performance interacts with Prin1 in children with incidences of SR compared to those without SR on the task. In the 21 children who showed no SR on any trial of MSTT, no consistent linear relationship was found between rank of MSTT performance and score on Prin1 (see Figure 2). By contrast, in the 22 subjects who responded with at least one example of SR, performance on MSTT correlated significantly with Prin1 outcomes (*p* < 0.0001). This relationship is apparent in Figure 3.

### Contributions to relationship between MSTT and linguistic performance variables

Partial CA was implemented to further explore the role of auditory discrimination (i.e., auditory phonological processing) and rhythm in the relationship between MSTT performance with Prin1 values, and outcomes for reading speed, reading accuracy, and word writing.

- Auditory discrimination: The C&S battery contains four tests which target auditory discrimination/auditory phonological processing: auditory word discrimination, alliteration, rhyme detection, and writing pseudowords. In the full data sample (*n* = 43), correcting for performance on these four tests substantially reduced correlation of MSTT with Prin1 [0.523 (*p* < 0.0001) to 0.259 (not significant)], reading speed [0.441 (*p* < 0.01) to 0.092 (not significant)], reading accuracy [0.603 (*p* < 0.0001) to 0.405 (*p* < 0.05) (significant)], and word writing [0.483 (*p* < 0.01) to 0.124 (not significant)]. This correction thus accounts for the significant relationship to MSTT to all of these variables except reading accuracy. Repeating this analysis with only the 22 subjects who exhibited SR showed a similar reduction with Prin1 [0.809 (*p* < 0.0001) to 0.412 (not significant)], reading speed [0.699 (*p* < 0.001) to 0.119 (not significant)], reading accuracy [0.759 (*p* < 0.0001) to 0.553 (*p* < 0.05) (significant)], and word writing [0.639 (*p* < 0.01) to 0.334 (not significant)].
- Rhythm production: No significant reduction was observed when correcting for rhythm production skills for the full data set or the subset of children who exhibited SR.

### MSTT error analysis

The significant amount of SR on MSTT calls into question whether characteristics of specific trials throughout the task, such as sequence order, were more likely to result in SR. Individual sequences contained a range of 1–3 stimulus changes. Only two trials contained one stimulus change (OOII and IIOO), and all of the rest contained two stimulus changes with the exception of one trial with three changes (OIOI). Trials with only one stimulus change resulted in fewer errors (19.7 versus 42.2% for sequences with more than one stimulus change) within the entire sample, suggesting an overall chunking effect (e.g., Baddeley, 2000). Error patterns throughout MSTT in children with SR were also compared to children without SR. Children without SR showed lower average error rates (8.3%) for sequences with one change compared to sequences with more than one change (26.8%). Children with SR showed the same pattern but higher overall error rates (30.7% for sequences with one versus 56.3% for sequences with more than one change). It is important to note that children with SR not only gave SR but also showed other types of errors (such as reversals) similar to the children without SR. We also performed a repetition analysis: as described above, MSTT comprised of nine unique sequences, presented twice in the same order with one sequence repeated another two times for a total of 20 trials. Performance on the first nine trials was compared with the repeated sequence, to investigate repetition effects between the groups. Errors were consistent throughout the task in children with no SR, demonstrating the same amount of error in the first presentation of all sequences as the repeated portion. By comparison, the rate of SR in children with SR increases from 21.8% in the first half to 29.1% of trials exhibiting SR in the repeated section. However, the rate of other errors (with no SR) in children with SR decreased from the first presentation to the repeated section (from 34% to 18.2%).

## Discussion

Our results indicate that there is a significant relationship between the MSTT and linguistic abilities. Our analysis revealed that performance on MSTT relates specifically to reading ability and phonological processing, which is consistent with previous studies that have found correlations between music perception/training and reading and phonological processing skills (e.g., Barwick et al., 1989; Lamb and Gregory, 1993; Anvari et al., 2002; Peynircioglu et al., 2002; Dege and Schwarzer, 2011; Moritz et al., 2012). In order to reduce our number of variables and to take into account the intercorrelations among them, we performed a PCA. Prin1, the first variable computed by our PCA, accounted for 33% of the performance variance in our cognitive-linguistic psychometric battery and showed a significant relationship to MSTT across the children as a group. Highest Loadings for Prin1 were found for reading measures including reading accuracy, reading speed, and word writing. Interestingly, an unexpected high incidence of extra responses on MSTT, which we term SR, was observed in half the subjects we tested and allows for a more detailed understanding of respective relations to the cognitive-linguistic psychometric battery. When analyzing children with and without SR separately, the two groups differed significantly on a number of measures including reading speed and phonological processing, and only children with SR showed a significant correlation between Prin1 and the music task. Furthermore, we could show that subtests targeting auditory discrimination and auditory phonological processing show a strong contribution to the observed relationships between MSTT and Prin1 in the full data set and in a subset of children who show SR.

Overall, our results are in line with previous results showing a relationship between music perception or musical expertise and language skills (Atterbury, 1985; Lamb and Gregory, 1993; Anvari et al., 2002; Peynircioglu et al., 2002; Besson et al., 2007; Thomson and Goswami, 2008; Moreno et al., 2009; Dege and Schwarzer, 2011; Huss et al., 2011; Moritz et al., 2012). Several research labs have reported altered language processing in professional musicians, suggesting for example that early phonetic processing is differently organized depending on musical expertise (Ott et al., 2011) or demonstrating perceptual advantages in musicians for the neural encoding of speech (e.g., Strait et al., 2011). Strait et al. (2011) assessed auditory working memory, attention, music aptitude, and neural sensitivity to acoustic regularities in school-age children with a variety of reading abilities. The authors report that music aptitude and literacy measures both show a relationship with working memory and attention as well as with the extent of subcortical adaptation to regularities in ongoing speech. In general, our findings further support these previous results and the theory that there may be common brain mechanisms utilized for language, reading, and music skills (see the OPERA hypothesis by Patel, 2011) that go beyond primary auditory processing and that these mechanisms may be characterized by how the nervous system responds to regularities in auditory input. However, we only observed a positive relationship between MSTT and Prin1 in children who showed SR. One could hypothesize that these children have problems with tracking regularities in the auditory input, problems with auditory sequencing, or perceiving the unique elements of the “auditory gestalt” for both language and music and that this is reflected by the correlation between performance on the music task and our PCA component Prin1. Furthermore, our error analysis showed that children with SR attended well to the task and showed a repetition effect throughout the task resulting in less non-SR errors (e.g., reversals) in the second half of the task. Nevertheless, the number of SR errors did not decrease throughout the task (but slightly increased), which seems to show that this deficit is more fundamental and probably related to perception/processing of the auditory input and seems to worsen over the time course of the task administration. Additionally, one needs to note children without SR showed ceiling effects on a variety of the tasks, which may have masked any correlation between MSTT and Prin1. Further studies should examine how our MSTT task is related to the underlying mechanisms for processing regularities in auditory input in typically and atypically developing children. Overall, ample evidence suggests that several music perception tasks relate to a variety of linguistic abilities. Many different tasks may be useful for linguistic screening of preliterate children, such as perception of musical meter (Goswami et al., 2013), implicit chord processing (Tillman et al., 2000), or pitch processing (Moreno et al., 2009), which have been suggested to share underlying cognitive-linguistic mechanisms. Future investigation is needed to determine which tasks provide the most specificity and sensitivity for detecting language deficits, for it remains unknown which element of music perception relates most strongly to language impairment (e.g., sequencing, rhythm, pitch discrimination).

Several research groups have already suggested impaired processing of auditory input in children with language or reading impairments (e.g., Tallal, 2004; Goswami, 2011), although the nature of these auditory processing difficulties has been highly debated. The ability to process brief, rapidly successive tones that require sequencing has been found to be a reliable indicator of language and reading levels in adults and children (Tallal, 1980; Kraus et al., 1996; Witton et al., 1998; Stein and Talcott, 1999; Ahissar et al., 2000; Banai et al., 2005), and a developmental predictor from infancy to future language skills and verbal intelligence (Benasich and Tallal, 2002; Benasich et al., 2006). Goswami (2011) recently proposed a temporal sampling framework for reading disabilities (developmental dyslexia). This framework suggests that individuals with RD have deficits with low-frequency phase locking mechanisms in auditory cortical areas, which then affects the development of phonological processing. Goswami (2011) argues that a general difficulty in distinguishing different modulation frequencies may affect syllable segmentation since it may lead to difficulties with tracking of the amplitude envelope. Rise times reflect the specific patterns of amplitude modulations that facilitate the temporal segmentation of the acoustic input into syllables and several studies have shown impairments of rise time perception in individuals with RD (Richardson et al., 2004; Hamalainen et al., 2005, 2008; Goswami et al., 2011; Beattie and Manis, 2012). Our results show that children with lower scores on reading, writing, and phonological tasks also demonstrated SR on MSTT, which seems to be consistent with the auditory sequencing impairment Tallal and colleagues reported in children with RD. However, it remains to be determined whether our MSTT task can be linked to rapid auditory processing or rise time perception or even to both or whether other cognitive processes such as auditory working memory or executive functioning may be involved.

The role of rhythm in reading ability is also a rapidly developing field of investigation (e.g., Thomson and Goswami, 2008; Moritz et al., 2012), given the inherent rhythmic element of speech production and prevalence among all languages (Cummins, 2002). The relationship between musical rhythm abilities and reading has yielded mixed correlational findings across tasks, ages, and perception versus production (Atterbury, 1985; Anvari et al., 2002; Montague, 2002; Overy, 2003; Moritz et al., 2012). In this study, we could not find a strong contribution of rhythm capability to the correlation between MSTT and linguistic processing capabilities. However, the rhythm performance capability tested in the battery only involves the skill of rhythmic pattern echoing. Future studies have to determine whether a more comprehensive rhythm test battery would show a stronger contribution which would support previous findings suggesting that the underlying auditory mechanism required to perform rhythmic tasks may be an important key element of literacy development, particularly in struggles with learning to read (e.g., Overy, 2003; Muneaux et al., 2004; Thomson et al., 2006; Huss et al., 2011).

It is also important to note that MSTT was designed in such a way that it avoids fine perception of pitch variations and therefore low performances on the musical task cannot be explained by low level deficits in the fine-grained pitch analysis (lesser than two semitones) that characterizes subjects with congenital amusia (Loui et al., 2011). Fine-grained pitch perception has been shown to be crucial for the perception and recognition of melodies, but not of words (Poeppel, 2001; Brattico et al., 2006). In contrast, pitch variations in speech are well perceived by congenital amusics because they are usually larger than half an octave (the pitch distance between two notes on the piano separated by twelve keys) (Hyde and Peretz, 2004). Therefore, MSTT was designed to preferentially (or perhaps selectively) involve mechanisms dedicated to patterned sequence processing, a basic mechanism which should be shared between music and language. As previously suggested, both the domains music and language can be thought of as patterned sound sequences hierarchically structured whose analysis requires the computation of inherent structural relations (e.g., Koelsch and Siebel, 2005; Osterhout et al., 2006; Fedorenko et al., 2009). Nevertheless, several alternative interpretations cannot be ruled out. For example, children with SR may have underlying auditory working memory problems or deficits in attention or executive functioning. Thus, the relationship between performance on the MSTT and executive functioning or working memory needs to be investigated in the future. However, children with SR do not exhibit lower performance on all administered tasks (only 7 out of 20), which would be expected if attention or executive functioning skills were impaired in this group.

Our study has several limitations that need to be taken into account when interpreting the results. First of all, we do not have any detailed information regarding the general non-verbal cognitive abilities of these children. Therefore, it remains unclear whether the observed findings may be explained by differences in general cognitive abilities (e.g., IQ or even overall processing speed). However, we examined the three subtests shape copying, figure order, and figure rotation and only observed differences in shape copying between children with and without SR. It should also be noted that shape copying, figure order, and figure rotation showed very low loadings on Prin1, the only Principle Component showing significant relationship to MSTT and therefore their contributions to the relationship between MSTT and linguistic variables should be minimal. Further studies need to examine the relationship between MSTT and a detailed battery of general nonverbal cognitive abilities. Furthermore, children with no SR are performing at ceiling on several of the C&S variables. While our results present a strong relation between MSTT performance and linguistic ability in children with SR independent of the non-SR group, the ceiling effects in our higher performing subjects leads to a difficult comparison between these groups, and the implications of MSTT as a useful screening tool for language ability. It is also important note that MSTT task has been administered in a classroom setting and therefore there may have been differences in the acoustic quality of the MSTT for different children. Nevertheless, the cognitive battery was administered individually to each child and therefore any confound is less likely. A further limitation of our current investigation is the inclusion of only children from upper-middle class families from a single school, so it remains unknown whether these findings would generalize to other socioeconomic levels or for children from different school settings.

Overall, performance on MSTT and its relation to linguistic abilities present results consistent with previous studies, suggesting a strong relationship between auditory discrimination, sequencing and phonological processing, and early literacy acquisition to music perception and discrimination (Tallal and Gaab, 2006). SR within MSTT has further revealed a possible deficit in identifying sequences of temporally segmented musical sounds, comparable to the perceptual demands of processing speech sounds in language. However, the underlying mechanisms for these auditory deficits need to be determined. Furthermore, investigation of children exhibiting SR demonstrates a significant relationship between SR and early reading and writing skills. More in-depth studies with children of different native languages and disabilities are necessary to understand the implications of the relation between SR and linguistic ability. Nonetheless, our MSTT design implemented within music class to a large group of children poses a convenient and feasible method of using this musical task to screen for difficulties in music perception. Future investigations will have to determine whether this task could be utilized as a diagnostic tool for the (early) identification of children with reading disabilities.

### Conflict of interest statement

The authors declare that the research was conducted in the absence of any commercial or financial relationships that could be construed as a potential conflict of interest.
